# Global Disease Detection—Achievements in Applied Public Health Research, Capacity Building, and Public Health Diplomacy, 2001–2016

**DOI:** 10.3201/eid2313.170859

**Published:** 2017-12

**Authors:** Carol Y. Rao, Grace W. Goryoka, Olga L. Henao, Kevin R. Clarke, Stephanie J. Salyer, Joel M. Montgomery

**Affiliations:** Centers for Disease Control and Prevention, Atlanta, Georgia, USA (C.Y. Rao, G.W. Goryoka, O.L. Henao, K.R. Clarke, S.J. Salyer, J.M. Montgomery);; Emory University, Atlanta (G.W. Goryoka)

**Keywords:** global disease detection program, GDD, infectious diseases, emerging infectious diseases, reemerging, global health, diplomacy, operations research, infectious disease outbreaks, public health surveillance, global health security

## Abstract

The Centers for Disease Control and Prevention has established 10 Global Disease Detection (GDD) Program regional centers around the world that serve as centers of excellence for public health research on emerging and reemerging infectious diseases. The core activities of the GDD Program focus on applied public health research, surveillance, laboratory, public health informatics, and technical capacity building. During 2015–2016, program staff conducted 205 discrete projects on a range of topics, including acute respiratory illnesses, health systems strengthening, infectious diseases at the human–animal interface, and emerging infectious diseases. Projects incorporated multiple core activities, with technical capacity building being most prevalent. Collaborating with host countries to implement such projects promotes public health diplomacy. The GDD Program continues to work with countries to strengthen core capacities so that emerging diseases can be detected and stopped faster and closer to the source, thereby enhancing global health security.

Infectious disease outbreaks present a serious health threat that requires early detection and effective preventive action to avoid regional or even global spread. Such actions enhance global health security by protecting the health of persons in the affected regions and in the United States. Recent epidemics, including severe acute respiratory syndrome (SARS) during 2002–2003, pandemic influenza A(H1N1) in 2009, Ebola virus disease in 2014, and Zika virus infection during 2015–2016, underscore this risk and highlight the critical need for building core global public health capacity for detection and response.

In 2001, the Centers for Disease Control and Prevention (CDC) established the International Emerging Infections Program (IEIP) to conduct applied public health surveillance and research aimed at preventing infectious disease outbreaks with pandemic potential. IEIP placed CDC staff in key overseas locations to work with national public health institutes and their partners to establish sentinel surveillance and conduct applied research on emerging infectious diseases. The program was modeled after the US-based Emerging Infections Program, a network of state health departments and their partners that conduct surveillance of certain infections and thereby provide a foundation for various epidemiologic studies to explore risk factors, spectrum of disease, and prevention strategies (*1*). IEIP had a similar objective but on a global platform; namely, to conduct applied public health research in strategic global locations to prevent, detect, and control emerging and reemerging pathogens.

CDC established the Global Disease Detection (GDD) Program in 2004 by using existing research programs within IEIP as the scientific backbone of its GDD regional centers; this effort was made in response to data gaps identified during the SARS epidemic. The GDD Program mission was to ensure that infectious diseases were detected and stopped at the source before crossing international borders (*2*). The GDD Program, like IEIP, set up a network of CDC technical experts stationed in GDD regional centers located in multiple countries across the World Health Organization (WHO) regions. GDD regional centers were initially set up in countries with IEIP presence (Thailand, Kenya, Guatemala, Egypt, China, and Kazakhstan). Subsequently, new GDD regional centers were established in Bangladesh, India, South Africa, and Georgia (*3*). These centers serve as regional resources for neighboring countries and are a framework for improving public health and global health security through close collaboration with local partners. To date, the 10 GDD regional centers have supported ≈90 countries around the world, including the United States (Figure). The GDD regional centers have assisted US domestic public health institutions in response to infectious diseases that affected international visitors while in the United States and US citizens while abroad.

**Figure:**
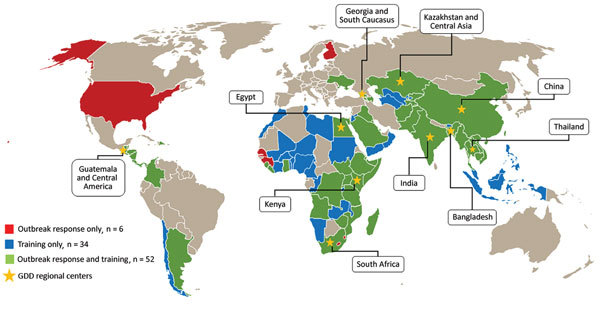
Geographic range of technical support provided by Global Disease Detection Program regional centers, 2006–2016. GDD, Global Disease Detection.

The GDD Program promotes intersectoral public health responses and applied epidemiologic research that include ministries of health and agriculture, academic institutions, other US government programs, and international and nongovernmental organizations. These established and trusted relationships with national governments enable more effective prevention and detection of emerging infectious diseases. The GDD regional centers also provide an in-country infrastructure that enables CDC to respond rapidly to public health threats. A critical strength of the GDD Program is the long-term assignment (i.e., 2–6 years) of epidemiologists, laboratorians, statisticians, and other diverse technical staff at GDD regional centers in host countries. The GDD technical staff work alongside locally hired technical staff to foster close collaboration and bilateral knowledge transfer with host country partners. These strategically placed GDD technical staff can have localized information for early detection of unusual infectious disease events. During public health emergencies, where time lost often equals lives lost, the ability to leverage trusted international public health scientific partnerships is essential for life-saving action. GDD field staff are often a first line of response during an epidemic. During the 2014–2016 West Africa Ebola outbreak, ≈30 GDD field-assigned staff, including US and local personnel, deployed from GDD regional centers to assist with establishing diagnostic, contact tracing, and data analysis capacity. GDD field staff’s experience in international settings was critical to the response and facilitated quick integration into ongoing response and prevention efforts. GDD’s sustained capacity-building efforts enabled these forward-deployed assets to respond quickly not only in their own regions but also across the globe.

## Activities and Accomplishments

The core activities of the GDD Program focus on applied public health research, surveillance, laboratory, public health informatics, and technical capacity building. Applied public health research refers to activities that generate data to answer a research question; test a hypothesis; evaluate a program or programmatic element (e.g., a public health practice, a surveillance system, data quality); or provide information for evidence-based decision making. Surveillance refers to activities that collect health-related data in a systematic manner over time to inform public health action. Laboratory refers to activities that collect specimens for laboratory analyses. Informatics refers to any activity that collects and aggregates data (paper-based or electronic) that could be used for further analysis. Capacity building refers to activities that increase the skills, infrastructure, or resources of individuals or partnering organizations. Current GDD Program projects incorporate multiple core activities, with technical capacity building, laboratory, and public health research being most common (Table 1). These activities are essential for the identification of new health threats, monitoring and tracking of health threats over time, and for conducting applied research and pathogen discovery. At times, regional centers might conduct studies of noninfectious causes of illnesses because it is not always clear whether the etiologic agent is a pathogen, a toxin, or some other cause at the beginning of an outbreak (*4*,*5*). The GDD Program also provides a robust framework for public health diplomacy and the development and implementation of coordinated multisite activities and studies (*6*).

**Table 1:** Number of projects conducted by Global Disease Detection Program regional centers, by activity type and year center was founded, fiscal years 2015 and 2016*

Year center founded	Country/region	No. projects	Activity type				
			PHR	S	L	PHI	CB
2004†	Thailand	20	8	13	11	13	7
2004	Kenya	36	12	10	9	9	19
2006	China	14	10	5	8	10	8
2006	Egypt	11	3	2	3	3	11
2006	Guatemala and Central America	22	15	11	14	15	10
2008	Kazakhstan and Central Asia	13	3	2	4	2	13
2009	India	13	4	4	5	3	13
2010	South Africa	28	9	5	12	5	20
2011‡	Bangladesh	31	22	14	17	1	11
2012	Georgia and South Caucasus	17	6	9	12	11	13
Total no. projects		205	92	75	95	72	125

*October 1, 2014–September 30, 2016. Activities do not sum across the rows because activity types are not mutually exclusive. CB, technical capacity building; IEIP, International Emerging Infections Program; L, laboratory; PHI, public health informatics; PHR, applied public health research; S, surveillance.  †IEIP-Thailand founded in 2001. ‡IEIP-Bangladesh founded in 2008.

## GDD by the Numbers

In 2015, the GDD Program performed a portfolio review of activities in the 10 GDD regional centers for fiscal years 2015 and 2016 (October 1, 2014–September 30, 2016). The unit of analysis was a GDD Program–funded project. Multiyear projects were counted once. Projects were not weighted by the size or scope of a project; thus, a small research study was equivalent to a large, multiyear, population-based surveillance project. We excluded projects that listed HIV (n = 2) or noncommunicable disease (n = 1) as their primary focus. We classified projects into core activity areas: technical capacity building, surveillance, applied public health research, laboratory, and informatics. Activity areas were not mutually exclusive, so a project could be classified in multiple areas.

Overall, the 10 GDD regional centers engaged in 205 discrete projects during October 2014–September 2016 (Table 1). The number of projects per GDD regional center ranged from 11 to 36. The variability in number of projects per center was attributable to a combination of factors, including the age of the center, the geographic region covered by the center, and funding and staffing resources available for the center. Capacity-building projects (n = 125) were most common (Table 1). We also classified technical projects into topical areas based on the key focus of the project. Topical areas were collated and categorized by major groupings (Table 2). The variability in the range of topical areas was attributable to a combination of factors, including the epidemiology of the disease (nationally and globally); available funding; the technical capacity at the local level; and the changing priorities of the United States and local partners (e.g., ministries of health, national public health institutes, and research institutes). Of 205 projects with a defined topical area, 24% (n = 50) were focused on acute respiratory illness (Table 2), which is expected given that respiratory disease surveillance has been a core function since the inception of the program. Health system strengthening (n = 36), One Health (n = 30), and emerging infectious disease (n = 22) were the next most common topical areas. The increasing prevalence of these new topical areas indicates an expansion of the breadth of projects being conducted by GDD regional centers.

**Table 2:** Number of projects conducted by Global Disease Detection Program regional centers, by topical area and activity type assessed, fiscal years 2015 and 2016*

Topical area	Definition	No. projects	Activity type*				
			PHR	S	L	PHI	CB
Acute respiratory illness	Syndromic surveillance focusing on respiratory pathogens (e.g., influenza, severe acute respiratory infections, pneumonia)	50	37	25	32	24	19
Health system strengthening	Incorporating any components of training, guidelines and protocol development, or capacity building to enhance the national disease surveillance system, workforce development, epidemiologic research, or information systems	36	7	2	7	6	34
One Health	The intersection of animal and human health, zoonotic diseases, or program development around zoonoses	30	13	14	15	10	16
Emerging infectious disease	Emerging or reemerging infectious disease within the regional center (e.g., hepatitis in Egypt and Georgia, polio in Kenya, neglected tropical diseases in Guatemala)	22	8	12	13	10	12
Emergency preparedness and response	Emergency preparedness and response efforts focusing on risk communication, pathogen detection, and outbreak investigation	19	2	1	0	2	19
Vectorborne infections	Vectorborne infections (e.g., malaria, dengue, Japanese encephalitis, Crimean-Congo hemorrhagic fever)	12	5	5	7	4	5
Hospital-associated infections	Healthcare infection and control	9	5	4	4	1	4
Tuberculosis	Tuberculosis infection, case findings, control, and treatment	9	7	1	5	4	3
Enteric disease	Diarrheal diseases or infection	8	3	6	6	6	6
Antimicrobial resistance	Antimicrobial drug–resistant pathogens	6	1	1	2	1	5
Acute febrile illness	Syndromic surveillance focusing on acute febrile or neurologic illness	4	4	4	4	4	2
Total no. projects		205	92	75	95	72	125

*October 1, 2014–September 30, 2016. Activities do not sum across the rows because activity types are not mutually exclusive. CB, technical capacity building; L, laboratory; PHI, public health informatics; PHR, applied public health research; S, surveillance.

## GDD Core Activities

### Applied Public Health Research

The GDD Program has a broad portfolio of applied public health research and special epidemiologic studies, ranging from ensuring infection control practices for Nipah virus in Bangladesh to evaluating antimicrobial drug–resistant invasive salmonellosis in Thailand (Table 3). Conducting applied public health research and epidemiologic studies in international settings can address important knowledge gaps in infectious disease issues. Many of these issues would be difficult to examine in the United States, primarily because of low prevalence of many infectious diseases. International public health research studies contribute to the scientific knowledge base and help answer questions that can influence US public health policy. Examples range from gathering data for the issuance of travel notices to conducting vaccine studies needed to guide domestic vaccination guidelines (*7*,*8*).

**Table 3:** Selected ongoing projects presented at the Global Disease Detection Program annual science meeting, by country and activity type assessed, June 2016, Atlanta, Georgia, USA*

Country	Title of presentation	Activity type					
		PHR	S	L	PHI	CB	
Bangladesh	Ensuring infection control is feasible and acceptable: identifying high-intensity interventions for Nipah-like illness and low-intensity interventions for routine use in Bangladesh	X			X	X	
	Making the case for rotavirus vaccination in Bangladesh: surveillance impacting public health interventions	X		X	X		
	Spatial heterogeneity for dengue risk in Bangladesh: significance for other arthropodborne infections such as Zika	X		X	X		
China	Verification of patients reported as central line–associated bloodstream infections (CLABSI) in a healthcare-associated infections surveillance system evaluation in Beijing	X			X		
	Risk factors for *Vibrio parahaemolyticus* infection in a southern coastal region in China	X			X		
Egypt	National surveillance of healthcare-associated infections and antimicrobial resistance in Egypt		X	X	X		
	Overview of GDD Egypt’s population-based syndromic surveillance—Damanhur, Egypt, 2009–2016	X	X	X	X		
	*Rickettsia typhi* as an underrecognized cause of acute undifferentiated febrile illness—Damanhour, Egypt, 2010–2014	X			X		
Georgia	Bloodborne disease prevalence in the blood supply, Georgia, 2012–2014	X			X		
	Hepatitis C elimination in Georgia: a one-of-a-kind program providing a golden opportunity to strengthen public health systems	X		X	X		
Guatemala	Influenza-like illness and influenza vaccination during pregnancy in Quetzaltenango, Guatemala	X	X	X	X		
	Participatory development of a congenital Chagas disease screening strategy after the vector control attack phase in Guatemala	X		X	X		
India	Acute encephalitis syndrome in Assam, India: importance of Japanese encephalitis in the adult population, 2014–2015	X		X	X		
	Redrawing the boundaries of Kyasanur Forest Disease (KFD) in India: early results of GHSA-supported acute febrile illness surveillance	X		X	X		
Kazakhstan	Strengthening the capacity of the Republic of Uzbekistan to combat antimicrobial resistance		X		X		
	Implementation of the CCHF surveillance enhancement activities in Kazakhstan, 2012–2015				X	X	
Kenya	Epidemiology of brucellosis and MERS-CoV in linked human and animal populations in Kenya	X		X	X		
	Indirect effects of 10-valent pneumococcal conjugate vaccine (PCV10) against adult pneumococcal pneumonia in rural western Kenya	X	X	X	X		
South Africa	Application of a simple differential diagnostic tool for solving febrile, neurologic and heamoragic fever cases in Southern Africa			X	X		
	Decline in syphilis seroprevalence among females of reproductive age in Northern Cape Province, South Africa, 2003–2012: utility of laboratory-based information	X			X		
Thailand	Spotted fever group, typhus group rickettsioses and Sennetsu neorickettsiosis in rural Thailand	X		X	X		
	Enhanced surveillance for severe pneumonia, Thailand 2010–2014		X	X	X		
	Epidemiology and antimicrobial resistance of invasive salmonellosis, rural Thailand, 2006–2014	X		X	X		
No. presentations by activity type		18	6	15	23	2	

*CB, technical capacity building; CCHF, Crimean-Congo hemorrhagic fever; GDD, Global Disease Detection; GHSA, Global Health Security Agenda; L, laboratory; MERS-CoV, Middle Eastern respiratory syndrome coronavirus; PHI, public health informatics; PHR, applied public health research; S, surveillance.

GDD regional centers work closely with the international partners, often a ministry of health or national public health institute, to identify common areas of research interests and national priorities. The data generated from these collaborations have been used by host governments to quantify the public health issue and, ultimately, to guide and inform public health policy. Implementing high-quality research studies also serves as a hands-on training mechanism for international partners. Projects are conducted in collaboration with the in-country hosts, from developing the concept, writing the research protocol, implementing the study, analyzing and interpreting the data, and publishing the results. A tangible way that highlights the results of these collaborations is dissemination of findings in the scientific literature. Since the inception of the GDD Program, GDD staff have authored or coauthored ≈875 peer-reviewed scientific articles (*9*).

### Surveillance

GDD regional centers partner with host countries to develop and strengthen surveillance for key illnesses and to limit spread of disease to the point of origin. Projects integrate laboratory, clinical, and epidemiologic information that can guide public health interventions and other control measures. GDD centers achieve this objective through several types of surveillance strategies, such as syndromic, laboratory-based, population-based, and sentinel systems (*10*–*15*). Population-based surveillance provide a framework for applied public health research that can help to characterize the burden, risk factors, and transmission characteristics of new or emerging infectious diseases and to assess the effectiveness of prevention strategies (*3*). Sentinel surveillance in a few key sites/facilities for specific or syndromic infectious diseases can help to identify emerging or reemerging pathogens (*16*).

Outbreaks of SARS and avian influenza A(H5N1) highlighted the need to have systems in place for detecting emerging pathogens (*3*). Thus, establishing population-based infectious disease surveillance for pneumonia and acute respiratory infections was a primary goal of the GDD Program (*3*). The resulting surveillance activities also provide a platform for other GDD core activities. Moreover, the GDD respiratory surveillance research projects have helped quantify burden of illness for pneumonia and influenza-associated acute respiratory illness, especially among children, and a high incidence of several respiratory pathogens, including respiratory syncytial virus, parainfluenza, and adenoviruses (*6*,*11*,*12*,*17*–*23*).

As GDD regional centers have matured, existing surveillance platforms have increasingly been adapted to include emerging pathogens, special noncommunicable disease studies, and projects focused on the animal–human interface (i.e., zoonotic diseases) (*24*–*28*). In 2014, the GDD regional centers began efforts to link common acute febrile illness (AFI) syndromic surveillance strategies across 5 regional sites (Egypt, Guatemala, India, Kenya, and Thailand) to gain a global perspective on AFI. Conducting AFI surveillance at GDD regional centers is of public health importance because AFI represents a common clinical syndrome for multiple diseases of outbreak potential or emerging zoonotic infections and provides an opportunity to evaluate novel diagnostics. Unlike respiratory illness syndromes such as severe acute respiratory illness and influenza-like illness, no international consensus case definition exists for AFI surveillance, although recommendations for improving methods have been proposed (*29*,*30*). In addition, very few published AFI etiology studies have been conducted in multiple countries. A literature review currently under way has found that, of 169 AFI studies aiming to identify etiology and published during 2005–2016, only 6 (4%) had enrolled cases in multiple countries (G. Kharod and C. Rhee, unpub. data).

A multisite research effort has the potential to catalyze historically disparate AFI syndromic surveillance systems toward globally comparable data of high utility at all levels for public health response. Network activities across different GDD regional centers that represent diverse disease risks enhanced the ability to study a range of infectious diseases for which a single country might not have the capacity or incidence of disease to study for evidence-based public health decision making. The GDD effort, to date, has included consistent case definition use with a focus on undifferentiated AFI, multipathogen detection of local and globally significant infectious diseases, use of standard and investigational diagnostics where feasible, and prospective sentinel health facility–based surveillance methods of >1 year in duration to evaluate seasonal epidemic trends. Barriers to launch and harmonization to a common research protocol have included variation in local priority pathogens, resource availability, and time required for integration into existing public health surveillance and healthcare networks. Established enhanced AFI surveillance has thus far provided a useful platform for investigating emerging infections with a febrile illness component, such as Zika virus and scrub typhus.

### Laboratory

Effective public health requires close collaboration between epidemiologists and laboratory scientists. GDD works with partner countries to strengthen diagnostic technical capacity for priority diseases; evaluate new laboratory diagnostics; establish frameworks for national laboratories that include quality assurance and specimen referral systems; improve biosafety/biosecurity; and train laboratory personnel on benchtop skills, laboratory management, and public health laboratory functions. These efforts have improved the capacity of GDD host countries and their regions to detect and respond to emerging infectious disease threats and to sustain these efforts through a strong cadre of laboratory scientists dedicated to improving the global public health laboratory infrastructure (*31*).

Research at the GDD regional centers has assisted in the detection and identification of 12 novel strains and pathogens that were new to the world and 62 novel strains or pathogens that were new to the region where they were discovered (*9*). GDD laboratorians have helped implement capacity to conduct >380 new diagnostic tests in 59 countries, improving disease detection capability and contributing to faster response times within the region.

### Public Health Informatics

Informatics is the application of public health information systems to capture, manage, analyze, and use information to improve public health practice (*32*). Examples of the key activities include the use of electronic databases, either as the source of data or as a method to collate data, for expediting the time between data collection and use. At GDD regional centers, public health informatics is a cross-cutting activity for disease surveillance, laboratory studies, and applied epidemiologic research to ensure that data are collected and managed in a systematic and reliable manner. Most GDD data-collecting projects currently under way have an informatics component (Table 3).

### Capacity Building

Strengthening the local public health capacity and workforce are key for improving the detection and response to infectious diseases globally. The transfer of epidemiology, laboratory, and emergency preparedness skills to local public health professionals is necessary for sustainability, both nationally and across regions. Capacity building is another cross-cutting activity at the GDD regional centers and ranges from establishing or strengthening existing surveillance, laboratory, emergency preparedness, and health systems to conducting high-quality epidemiologic research studies to address knowledge gaps. This capacity is achieved through on-the-job training of local partners, providing technical expertise, conducting high-quality research studies, and collaborating on analysis of information to inform evidence-based decision making.

## Public Health Diplomacy

Scientific exchange can play a strong role in building bonds across countries. Because health is an area of concern for all nations, international projects that address a common threat, such as infectious diseases that easily cross borders, can open avenues of communication and ease tensions between the United States and other nations (*33*). GDD China serves as an example of how 2 strong national public health institutes (1 in China and 1 in the United States) can collaborate and benefit. During the West Africa Ebola outbreak in 2014, China CDC had the resources and willingness to respond but not necessarily the US CDC experience or technical expertise with Ebola outbreaks and response. Since 2006, Chinese laboratorians have worked alongside US colleagues to build greater diagnostic testing capacity throughout China. Because of this preexisting relationship, the 2 countries were able to forge a new type of collaboration in Sierra Leone; scientists from both countries worked together to offer critical training and resources to Sierra Leone to help stop the spread of the largest Ebola outbreak in history (*34*). By building strong partnerships and scientific systems, GDD protects the United States and countries around the world from threats to health, safety, and security.

## Lessons Learned and the Future

The GDD Program promotes the prompt detection and mitigation of disease threats globally. GDD works with multiple countries (Figure) to conduct applied public health research and develop and enhance public health capacity to rapidly detect, accurately identify, and promptly contain emerging and reemerging infectious diseases. The activities of the GDD Program are critical to help countries improve their disease surveillance networks and enhance laboratory capabilities for detection of emerging pathogens. The program also has greatly expanded epidemiology workforce networks to meet their commitment to global health security and the International Health Regulations 2005.

The activities of the GDD Program have developed needed technical capacity, advanced science, and provided critical information for policy change. Activities of the GDD regional centers have allowed a greater understanding of what infections or conditions are of concern in the countries and regions in which they work. They have increased awareness of the emergence of antimicrobial resistance and the growing threat of infections that can be acquired in healthcare settings (*35*,*36*). Strengthening disease surveillance, applied public health research, and laboratory capacity have allowed for a better understanding of pathogens associated with illnesses that present with acute fever. The activities established serve as a base or launch pad for the rapid and timely implementation of surveillance for emerging infections like Zika virus and applied epidemiologic research studies to better understand which populations are being affected and to enumerate potential factors associated with infection and spread of illness.

As new laboratory techniques for the detection of pathogens are developed, the GDD regional centers have served as a platform to examine the performance of these new tests in multiple settings and promote the adoption of the new techniques in multiple countries. Because of ongoing surveillance and routine collection of epidemiologic information, GDD regional centers and the countries they work with have the tools needed to best characterize pathogens that are circulating and explore potential reservoirs and sources associated with these infections. Increased informatics capacity is concurrently enabling the active linkage of information and interfacing of data housed in multiple data systems within the countries and regions.

GDD regional centers make critical contributions to global disease detection by improving infectious disease detection capacity through integration of applied public health research and laboratory capacity building, which in turn will generate quality data that can inform high-level policy. The GDD Program has matured and transformed over the past 10 years and continues to evolve. Further advancing the technical capacity that has already been developed is allowing the GDD Program to focus on needed research and generation of data to develop and evaluate interventions and inform policies needed to reduce burden of multiple conditions worldwide. Examples of research activities needed include studies to understand the actual burden of conditions at play, assessments of the impact of multiple conditions on local and global populations, quantification of the societal and economic costs of illnesses, and evaluation of control measures.

Threats posed by emerging pandemics and other infectious diseases will remain a challenge to global health security, endangering economies and decreasing political stability. GDD will continue to work with countries to strengthen core capacities and conduct applied public health research so that emerging and reemerging diseases and conditions can be detected and stopped faster and closer to the source, thereby enhancing global health security.
